# Ex-aspirated: A Case of Dental Product Aspiration With Retrieval Methodology and Current Review

**DOI:** 10.7759/cureus.39074

**Published:** 2023-05-16

**Authors:** Nicholas D Nassif, Manveer Ubhi, Aniruddh Kapoor

**Affiliations:** 1 Internal Medicine, Advocate Aurora Healthcare, Milwaukee, USA; 2 Pulmonary-Critical Care, Advocate Aurora Healthcare, Milwaukee, USA

**Keywords:** cough, dental esthetics, tracheal foreign body, rigid and fiber-optic bronchoscopy, bronchoscopy

## Abstract

Foreign body aspiration is of significant prevalence in the pediatric and young adult populations. After dental work, patients are more likely to develop pulmonary symptoms secondary to aspiration events within the tracheobronchial tree. Herein, we describe the clinical case of a 22-year-old man with a past medical history of epilepsy and tuberous sclerosis who presented to his primary care provider for chronic coughing and wheezing. With symptoms refractory to albuterol and control of allergies, radiography was obtained, revealing a 4.1 cm dental product in the right bronchus. We provide an overview of our retrieval method as well as a comparison of flexible and rigid bronchoscopies and the bronchoscopic tools available.

## Introduction

Foreign body aspiration (FBA) is a potentially life-threatening event. They are a common complication in children but are often only seen in adults with impaired levels of consciousness [1]. The extraction of these foreign bodies (FBs) can be quite challenging given the rigidity of the airway, the size and shape of the dental products, and the ability of the FB to be lodged in the tissue. There is limited epidemiological data; one study required 16 years of review to amass 43 adults with FBA [2]. The main risk factors include trauma, alcohol, drug use, and neurological disturbances (i.e., stroke, Parkinson’s disease, and seizures) [3]. FBA symptoms depend on the degree of obstruction and location. Typically, the symptoms of incomplete obstruction are non-specific and may include shortness of breath, cough with or without sputum production, and signs of tachypnea, stridor, or wheezing. Furthermore, an inciting event may or may not be reported with incomplete obstruction. Often, the patient will seek medical attention when complications from the obstruction occur, such as pneumonia. On the contrary, central airway obstruction may present with respiratory distress and may even precipitate cardiac arrest. Complications of FBA are numerous, including dislodgement of the FBA to a worsened position, post-obstructive pneumonia, atelectasis, and even pneumothorax, pneumomediastinum, and the need for a thoracotomy [4]. Depending on the FBA material, shape, and retrieval process, scarring and granulation tissue can form. Additionally, given the numerous shapes of FB that have been aspirated, there is no "one size fits all" in the retrieval process, allowing pulmonologists to use a myriad of tools during bronchoscopy. We present the case of a young man with a seizure history who aspirated a dental product and provide a summary of our management of the FBA and an in-depth review of available procedural methods.

## Case presentation

**Figure 1:**
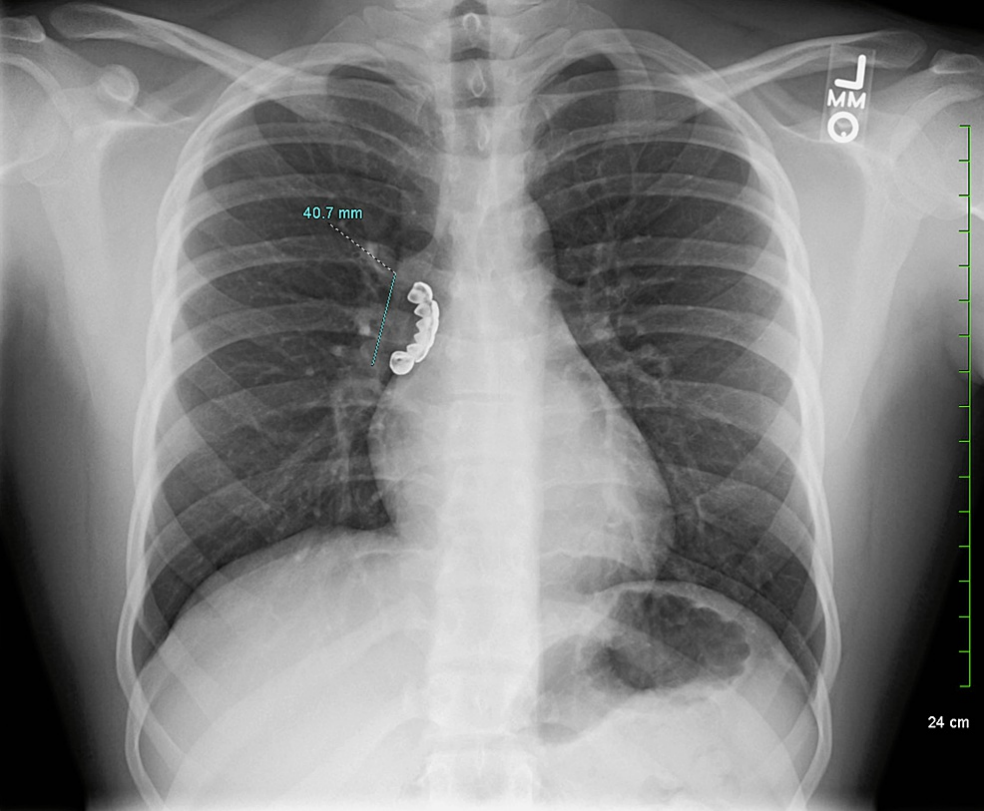
Initial chest radiography

This is a case report of a 22-year-old man with a history of epilepsy in the context of tuberous sclerosis. Initially, he sought outpatient care from his primary care provider for new concerns about a chronic, persistent cough lasting for several months. At first, he did not report any pulmonary symptoms aside from the cough, so conservative measures were utilized, including albuterol. Prior to the outpatient visit, he had consulted his neurologist for the management of his epilepsy, which was poorly controlled. In fact, the patient was experiencing approximately one seizure a week, and his epileptic regimen was being titrated. The cough persisted, prompting radiography. A chest x-ray revealed a radiopaque foreign body measuring about 4.1 cm over the right main bronchus (Figure 1).

**Figure 2:**
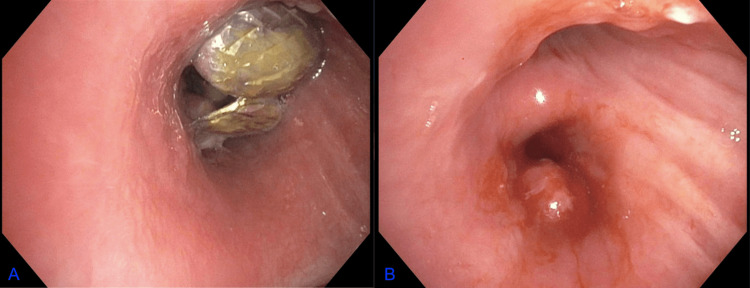
Bronchoscopic views (pre- and post-retrieval, respectively) A: right main stem bronchus with a foreign body visualized; B: right main stem bronchus with the foreign body removed

The FB appeared to be horseshoe-shaped, with sharp and jagged edges resembling teeth. Bronchoscopy was performed to remove the inhaled foreign body, as shown in the pre-and post-device retrieval images shown below (Figures 2A and 2B). The procedure is described below.

**Figure 3:**
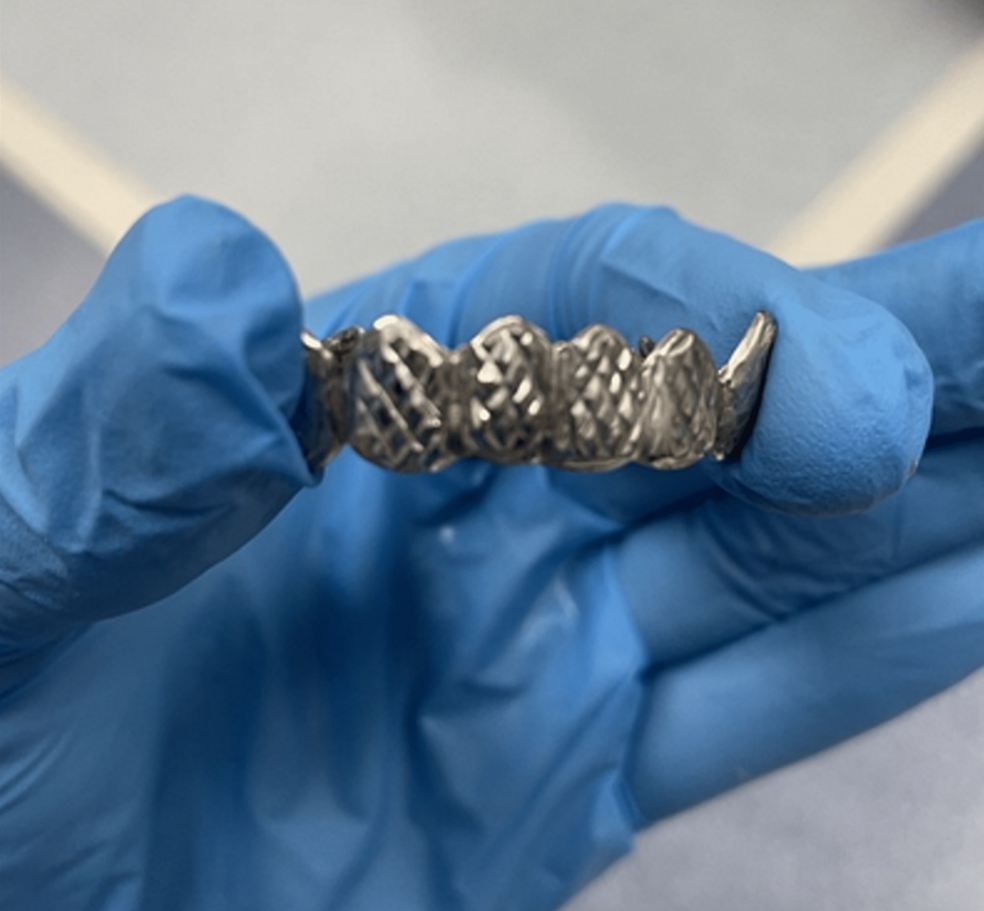
Dental device retrieved

Despite traversing the vocal cords, retrieval was particularly challenging given the shape and size of the dental product (Figure 3).

Unfortunately, the horseshoe-shaped object had sharp and jagged edges. Our primary concern was a vocal cord injury during retrieval. As such, we entertained several different extraction modalities, including flexible bronchoscopy, rigid bronchoscopy, and, as a last resort, cardiothoracic surgery. After extensive discussion, an initial attempt with a flexible bronchoscope was agreed upon. After intubation with an 8.0 endotracheal tube, a flexible bronchoscope was introduced into the airway. Initially, under direct visualization, toothed forceps were used to dislodge the dental device from the bronchial wall. The resultant minor bleeding was controlled by instilling chilled saline. After mobilization, the forceps were also used to "aim" the dental device so that the rounded shape would cross the vocal cords first. Unfortunately, the forceps were unable to provide enough grip to ensure safe retrieval. To ensure a tighter grip, a lasso was introduced and threaded over and around the dental device. Using the lasso, the dental device was pulled up to the bronchoscope. Then, simultaneously, the dental device, bronchoscope, and endotracheal tube were all removed. Initially, likely because of the size of the dental device, the patient did experience bronchospasm complicated by hypoxia, requiring reintubation. Fortunately, after steroid treatment, the patient was safely extubated and able to return home.

## Discussion

Management is dependent on acuity. If central airway obstruction is suspected, securing the airway is of paramount importance. Endotracheal intubation is performed by an experienced operator. Further, if available, additional pulmonary and/or surgical support may be advisable. Once stable, chest radiography, including X-ray (chest radiography (CXR)) and/or a computed tomography (CT) scan, is valuable in determining the etiology, extent, and site of obstruction. In our case, the patient presented to his primary care physician with increasing wheezing deemed secondary to asthma. CXR later revealed FBA.

After securing the airway, visualization, and retrieval of the FB by flexible bronchoscopy or rigid bronchoscopy are performed. Flexible bronchoscopy is often used for the diagnosis and extraction of FBs in those with conscious sedation in non-acute status [5]. However, in patients with large FBs with asphyxiation or structurally dangerous FBs (sharp, pointed), rigid bronchoscopy has demonstrated better outcomes [6]. Both bronchoscopes have advantages and disadvantages. Our approach is to attempt flexible bronchoscopy first. Flexible bronchoscopy is readily available and can be performed under moderate sedation. However, rigid bronchoscopy allows for continuous gas exchange and suctioning during active instrumentation and has a larger internal diameter (rigid internal: 6-8 mm, flexible internal: 5-6 mm). Consequently, this size discrepancy allows the flexible bronchoscope to access more distal airways, while the rigid bronchoscope is limited to first- or second-generation bronchi [7]. A comparison of the two interventions can be seen below (Table 1).

**Table 1:** Comparison of flexible and rigid bronchoscopy

Flexible bronchoscopy	Rigid bronchoscopy
Readily available	Limited availability
The internal diameter is smaller (5–6 mm)	The internal diameter is larger (6–8 mm)
Does not secure the airway (single lumen)	Secures the airway (double lumen)
Conscious sedation	Full sedation
Can reach distal airways	Limited to proximal airways
Indicated in cervical trauma patients	Contraindicated in cervical trauma patients

A pulmonologist’s toolbelt is ever-growing. The go-to device is typically the standard alligator forceps. Second, attempts can be made with a wired basket, advanced through the suction cannula, opened and rotated on an axis around the FB, locked, and then withdrawn [8]. Third, we attempted to use a push-device multiprong snare, which is often helpful with large foreign bodies [9]. Additionally, cryotherapy can be used to freeze the FB to allow for quick and easy removal [10].

The goal of this case report was to present a uniquely challenging case regarding foreign body retrieval and reflect on how we can approach similar cases in the future, yielding even better results. We hypothesize a history of epilepsy or impaired cognition as significant patient risk factors for FBA, and severe obstruction with FBA may warrant the utilization of rigid bronchoscopy to secure and maintain a patent airway throughout the prolonged retrieval process.

## Conclusions

Aspiration of dental foreign bodies into the tracheobronchial tree is an all-too-common occurrence. Given the myriad types, shapes, and sizes of dental devices on the market, the endoscopic pulmonary approach varies greatly. The goal of this case presentation is primarily to publicize our technique, shed light on the dangers of non-implanted dental devices, especially in those at elevated risk of aspiration or impaired levels of consciousness, and provide a thorough review of bronchoscopes and tools available for use.
